# Integration of lodging resistance QTL in soybean

**DOI:** 10.1038/s41598-019-42965-6

**Published:** 2019-04-25

**Authors:** Sadal Hwang, Tong Geon Lee

**Affiliations:** 1 Gulf Coast Research and Education Center, University of Florida, Wimauma, FL 33598 USA; 2 Horticultural Sciences Department, University of Florida, Gainesville, FL 32611 USA

**Keywords:** Agricultural genetics, Plant breeding, Quantitative trait

## Abstract

Poor lodging resistance could limit increases in soybean yield. Previously, a considerable number of observations of quantitative trait loci (QTL) for lodging resistance have been reported by independent studies. The integration of these QTL into a consensus map will provide further evidence of their usefulness in soybean improvement. To improve informative QTL in soybean, a mapping population from a cross between the Harosoy and Clark cultivars, which inherit major U.S. soybean genetic backgrounds, was used along with previous mapping populations to identify QTL for lodging resistance. Together with 78 QTL for lodging collected from eighteen independent studies, a total of 88 QTL were projected onto the soybean consensus map. A total of 16 significant QTL clusters were observed; fourteen of them were confirmed in either two or more mapping populations or a single population subjected to different environmental conditions. Four QTL (one on chromosome 7 and three on 10) were newly identified in the present study. Further, meta-analysis was used to integrate QTL across different studies, resulting in two significant meta-QTL each on chromosomes 6 and 19. Our results provide deeper knowledge of valuable lodging resistance QTL in soybean, and these QTL could be used to increase lodging resistance.

## Introduction

Lodging is a morphological trait that limits crop yield potential. For example, the effect of lodging decreased yield by approximately 40% in oat, a cereal crop^1^. In dicotyledonous plants, such as soybean, environmental conditions that promote high yield could aggravate lodging by stimulating the height and vegetative growth of the plants^2^. Moreover, lodging could reduce seed yield by up to 10%, suggesting that high-yielding germplasm may be particularly affected by yield loss^3,4^. The physiological mechanisms of yield loss under lodging remain unknown. A reduction in photosynthesis within a canopy may suppress the transport of water and photosynthetic assimilates^5–7^. Stem strength^8^, root morphology^9^, and node number^10^ can influence sensitivity to lodging.

Berry *et al*.^5^ demonstrated two types of lodging: root (bending-type) and stem (breaking-type) lodging. Root lodging occurs at the base of a plant due to a lack of anchorage strength. Stem lodging arises at any point on the stem due to weakened stem bending strength. The former is the most common type of lodging in crop plants, including soybean. The latter usually occurs at the lower portion of a plant when the plant starts grain or seed filling, making the stem dry and brittle.

Improved management practices such as disease control, plant density, planting date, and fertilizer application have been utilized to prevent lodging in soybean. A simulated study has shown that lodging at the growth stages R3, R5, and R6 reduces seed yield by 12–18, 18–32, and 13–15%, respectively^4^. High plant density can also aggravate lodging without increasing yield. Approximately 120,000 to 150,000 seeds/acre sown in 30-inch rows would be ideal to produce maximum yield in the Midwest region of the U.S.^11,12^. Pod or stem disease may easily spread among lodged plants. Early planted short-season soybean could be prone to lodging due to damage by the soybean stem borer^13^. Lodging can also slow down the harvesting process because it is not easy to cut and gather lodged plants into a combine.

The heritability of lodging resistance and its relationship with plant height or seed yield have been evaluated. In a wide range of population studies, a moderate to high heritability of lodging resistance has been reported^14–21^. There is evidence that lodging and height are positively correlated^22–26^. However, lodging and seed yield showed the following discrepancies in the sign of their correlation: not significant^27^, positive^23,26,28–31^, negative^25,32^, and positive or negative^25,33^. These reports suggested that seed yield could be weakly affected by indirect selection on lodging.

Though intensive QTL analysis has been conducted for lodging resistance in soybean^19,22–26,31,33–37^, there has not been a comprehensive study that integrates data from a wide range of seasons and populations. A deeper knowledge of informative QTL has the potential to provide the community of soybean researchers with new tools such as a consensus marker for marker-assisted selection (MAS). Therefore, in the study below, we examine QTL and meta-QTL for lodging resistance across independent populations and a population that we created. We investigate statistically significant QTL-lodging associations and major QTL in the investigated populations.

## Results

### Development of the Harosoy x Clark genetic map

The genetic map of the Harosoy x Clark (henceforth referred to as H x C) population was constructed (Supplemental Fig. 1): the total genetic map distance was 3769 cM and the average map distance of the H x C population was 5.21 cM. Since the average map distance of the consensus genetic map was 0.41^38^, the map resolution of the H x C population was 12.5 (=5.21 cM/0.41 cM) times lower than that of the soybean consensus map.

### Analysis of the field data of H x C population

A parental difference would be a necessary assumption of a single-QTL model, since transgressive segregation requires at least two QTL. The differences between two parental means were tested by a *t*-test (H_a_: Two parental means are not the same) in the irrigated and rainfed trials (Table 1). Parental differences for lodging were statistically significant (*P* ≤ 0.01) under irrigated and rainfed conditions in 1998. The statistical power of each *t*-test in 1998 was greater than 0.8. The parental means in 1999 were not significantly different under either water condition.

**Table 1:** Population statistics for lodging in the Harosoy x Clark population.

Year			1998	1999	1998	1999
Water treatment			Irrigated	Irrigated	Rainfed	Rainfed
Parental	Mean	Harosoy	2.925	2.550	3.225	2.000
		Clark	2.625	2.500	2.125	2.225
	SD^a^	Harosoy	0.245	0.510	0.573	0.513
		Clark	0.393	0.628	0.358	0.734
	*t*-test^b^		**	ns	**	ns
	Power^c^		82.6	5.90	100.0	20.3
RIL	Mean		2.74^d^	2.80	2.87	2.29
	Minimum		1.00	1.00	1.00	1.00
	Maximum		4.50	4.50	5.00	4.25
	Skewness		0.126	0.364	0.242	0.372
Population	Kurtosis		0.801	−0.257	−0.059	0.328
	Normality^e^	S-W test	0.0061	<0.0001	0.0403	<0.0001
		K-S test	0.0220	<0.0100	0.1500	<0.0100
	*H* ^2f^			0.679		0.685
	CI of *H*^2^			0.595–0.746		0.602–0.750

^a^The SD represents the standard deviation of each parental line with 20 samples.

^b^The significance from a two-tailed *t*-test was presented as ** and ns, which indicates that *P* values were less than 0.01 and greater than 0.05, respectively.

^c^The statistical power was estimated by a two-tailed *t*-test.

^d^Plant lodging was visually assessed by using a score that ranged from 1 (erect) to 5 (prostrate).

^e^The *P* values are from two types of tests used to test the normality of progeny means for each water-year data set (S-W, Shapiro-Wilk; K-S, Kolmogorov-Smirnov).

^f^The 95% of confidence intervals (CIs) of heritability were estimated from the combined two-year data from the irrigated and rainfed field trials.

The single-QTL model in our study assumes that lodging follows a normal distribution, i.e., that the residuals follow a normal distribution and are independent. A normality test was performed for each water-year data set (Table 1). The average means of lodging ranged from 2.29 to 2.80. The minimum value of lodging was 1, and the maximum value of lodging was close to 5. The Shapiro-Wilk and Kolmogorov-Smirnov tests (H_a_: A random variable for lodging does not follow a normal distribution) showed that lodging did not follow a normal distribution. The Kolmogorov-Smirnov test indicated that lodging under rainfed conditions in 1998 followed a normal distribution. The degree of skewness was less than 0.5 and positive. Kurtosis was low under rainfed and irrigated conditions in 1998 and 1999, which indicated no acute peakedness around the distributional mean. Thus, we assumed that our field data for lodging followed a normal distribution. No attempt to improve normality was made in this study.

Based on the analysis of variance (ANOVA) result, the heritability of lodging resistance was estimated over years in each type of water treatment (Table 1 and Supplementary Table 1). While the variance for the Water x recombinant inbred lines (RIL) interaction was not statistically significant, it was (*P* < 0.0001) for RIL and the Year x Water, Year x RIL and Year x Water x RIL interactions (Supplemental Table 1). Since all interactions except for Water x RIL were significant, the RIL means were not averaged across the water and year treatments for subsequent combined-QTL analyses. Instead, the LS mean for each RIL was estimated from each water-year data set. The two heritability values under each water treatment were not substantially different, because the Water x RIL interaction was not statistically significant (Table 1). The heritability of lodging ranged from 0.595 to 0.750 within the upper and lower values of the 95% confidence intervals (CIs).

### QTL mapping of H x C population

The associations between lodging resistance and molecular markers were evaluated in the H x C population (Table 2). As we described earlier, although the parental means in 1999 were not different, we conducted QTL analysis for all field trials. A total of ten putative QTL were identified on chromosomes 7, 10, 13, 14, 15, and 18. Three of the ten QTL were identified on chromosome 10. Two QTL, Lg02 and Lg06, which were positioned at 99.69 and 94.97 cM, had overlapping CIs. The phenotypic variation (R^2^ value) explained by a QTL marker ranged from 0.04 to 0.27. Interestingly, two QTL markers on chromosome 10, BARC-050013-09288 and *E2*, showed the highest R^2^ value, suggesting that these QTL could make major contributions to the lodging resistance of the H x C population. Further, the *E2* marker showed the highest QTL effect, and the allele derived from Harosoy was detected in this locus.

**Table 2:** Lodging resistance QTL in the Harosoy x Clark population.

QTL	Chromosome	Year	Water^a^	QTL marker	QTL^b^	Favorable	R^2e^	QTL^f^	LOD	Flanking markers and their positions^b^
name	number				position	allele^d^		effect		95% CI^g^
Lg01	7	1998	I	Sat_288	72.83	Clark	0.04	0.13	3.3	BARC-017117-02201 - Satt551 65.88–89.45
Lg02	10	1998	I	BARC-015925-02017	99.69	Harosoy	0.10	0.22	8.4	BARC-050013-09288 - Satt153 94.97–106.32
Lg03	15	1998	I	BARC-057969-15031	77.04	Clark	0.09	0.19	7.0	BARC-053201-11762 - BARC-057969-15031 76.60–77.04
Lg04	18	1998	I	Sat_064	101.82	Clark	0.07	0.17	5.2	Sat_064 - BARC-057845-14952 101.82–103.11
Lg05	14	1998	R	BARC-065009-19043	56.60	Clark	0.07	0.19	4.4	BARC-065009-19043 - Satt474 56.60–63.36
Lg06	10	1999	I	BARC-050013-09288	94.97	Harosoy	0.25	0.40	86.1	Satt592 - Satt581 91.36–95.60
Lg07	13	1999	I	BARC-055613-13490	77.16	Harosoy	0.05	0.23	4.9	BARC-055229–13122 - Satt144 71.89–78.89
Lg08	18	1999	I	Sat_131	32.88	Harosoy	0.07	0.20	5.7	BARC-014395-01348 - Satt324 19.48–35.43
Lg09	10	1999	R	*E2*	121.41^c^	Harosoy	0.27	0.43	20.3	BARC-063361-18346 - BARC-041935-08142 120.36–122.45
Lg10	15	1999	R	BARC-058675-17461	68.06	Clark	0.07	0.21	3.9	BARC-050109-09389 - BARC-058675-17461 54.94–68.06

^a^Two different water treatments were applied to irrigated and rainfed field trials. I and R represent irrigated and rainfed field water conditions, respectively.

^b^QTL markers and flanking markers were positioned based on the Consensus 4.0 genetic map of soybean.

^c^Because *E2* was not positioned on the Consensus 4.0 genetic map of soybean, the nearest marker, BARC-024447-04891, was considered as the QTL marker of *E2*.

^d^Based on the maximum likelihood-estimated QTL positions, alleles with a low plant lodging score were defined as favorable alleles.

^e^The amount of phenotypic variation explained by a QTL marker was estimated as a R^2^ value.

^f^Additive effects were estimated as half the difference between the average effects of two parental alleles at the maximum likelihood-estimated QTL positions.

^g^The LOD values with ±1 deviation were used to estimate the 95% confidence invervals of the maximum likelihood-estimated QTL positions.

### Confirmation of QTL

**Figure 1:**
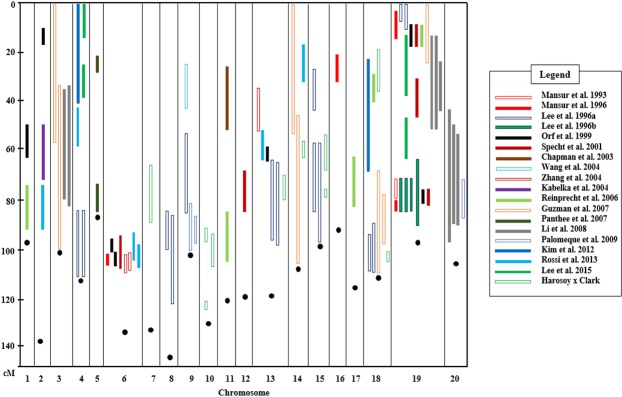
Integration of lodging QTL in soybean. After considering the confidence intervals of all QTL identified in this study and independent researches, significant QTL were projected onto the Consensus 4.0 genetic map of soybean. Black dots indicate the telomere-proximal end of each chromosome based on the consensus map.

Next, we integrated QTL from 19 previous studies and ours (Tables 2 and 3, and Supplemental Table 2). A total of 88 QTL (78 QTL from previous studies and 10 QTL from the H x C population) were projected onto the consensus genetic map of soybean based on 95% CIs to confirm QTL (Fig. 1). QTL identified along with overlapping CIs in two or more mapping populations were considered strongly confirmed QTL^39^. The QTL identified from different environmental conditions (such as location, year, and water treatment) and with overlapping CIs in only one population were considered weakly confirmed QTL^39^.

**Table 3:** Previous QTL mapping studies for lodging.

Reference	Population				QTL		
	Name	Size	Type	Cross type	Method^a^	Marker type	Number^b^
^82^	Minsoy x Noir 1	69	F_2:5_ RIL	*G*. *max* x *G*. *max*	IM	RFLP	1
^23^	Minsoy x Noir 1	284	F_7_-derived RIL	*G*. *max* x *G*. *max*	SMA	RFLP + SSR	4
^22^	Young x PI 416937	120	F_4_-derived RIL	*G*. *max* x *G*. *max*	SMA	RFLP	14
^78^	PI 97100 x Coker 237	111	F_2_	*G*. *max* x *G*. *max*	SMA + IM	RFLP + Classical	4
^33^	Minsoy x Noir 1	240	F_7_-derived RIL	*G*. *max* x *G*. *max*	IM	SSR + Classical	5
^33^	Minsoy x Archer	233	F_7_-derived RIL	*G*. *max* x *G*. *max*	IM	SSR + Classical	1
^33^	Noir 1 x Archer	240	F_7_-derived RIL	*G*. *max* x *G*. *max*	IM	SSR + Classical	1
^26^	Minsoy x Noir 1	236	F_7:11_ RIL	*G*. *max* x *G*. *max*	CIM	RFLP + SSR + Classical	5
^16^	Essex x Williams	177	F_4:6_ RIL	*G*. *max* x *G*. *max*	SMA	SSR	1
^36^	PI 468916 x IA2008	110	BC_2_F_4_-derived	*G*. *max* x *G*. *soja*	CIM	SSR	1
^10^	Kefeng No.1 x Nanong 1138-2	184	F_7:10_ RIL	*G*. *max* x *G*. *max*	CIM	RFLP + SSR	3
^31^	BSR 101 x LG82-8379	167	F_5_-derived RIL	*G*. *max* x *G*. *soja*	SMA	SSR	1
^25^	RG10 x OX948	169	F_6_ RIL	*G*. *max* x *G*. *max*	CIM	SSR	5
^18^	LG96–6607 x Lawrence^3^	94	BC_3_F_2_-derived	*G*. *max* x *G*. *max*	SMA	SSR	2
^18^	LG92-1143 x Beeson 80^2^	68	BC_2_F_2_-derived	*G*. *max* x *G*. *max*	SMA	SSR	2
^18^	LG94–1713 x Kenwood^1^	74	BC_1_F_2_-derived	*G*. *max* x *G*. *max*	SMA	SSR	3
^24^	N87-984-16 x TN93-99	101	F_6_-derived RIL	*G*. *max* x *G*. *max*	CIM	SSR	2
^37^	PI 245331 × 7499	147	BC_2_F_4_-derived	*G*. *max* x *G*. *soja*	SMA	SSR	8
^20^	OAC Millennium x Heinong 38	98	F_4:7_ RIL	*G*. *max* x *G*. *max*	SMA	SSR	2
^83^	Pioneer 9071 x Line 8902	133	F_4:7_ RIL	*G*. *max* x *G*. *max*	SMA	SSR	1
^34^	PI 436684 x PI 548557	116	BC_2_F_3_-derived	*G*. *max* x *G*. *max*	CIM	SNP	1
^34^	PI 90566-1 x Williams 82	93	BC_2_F_3_-derived	*G*. *max* x *G*. *max*	CIM	SNP	1
^84^	OAC Millennium x Heinong 38	92	F_4:7_ RIL	*G*. *max* x *G*. *max*	SMA	SSR	4
^84^	Pioneer 9071 × 8902	131	F_4:7_ RIL	*G*. *max* x *G*. *max*	SMA	SSR	2
^19^	PI 567310B x Wyandot	91	F_7_-derived RIL	*G*. *max* x *G*. *max*	SMA + CIM	SNP	4

^a^IM, interval mapping; SMA, single marker analysis; Single marker analysis was based on one-way ANOVA or a paired *t*-test between any two parental alleles.

^b^QTL markers from mapping study.

There were 16 QTL clusters on chromosomes 2 (one QTL), 3 (one), 4 (two), 6 (one), 8 (one), 9 (one), 10 (one), 13 (two), 14 (one), 15 (one), 18 (two), 19 (two), and 20 (one). Two QTL clusters on chromosomes 4 (telomere proximal) and 8 were identified in a single population in the same environment^22^ (data combined across locations). Therefore, the two QTL clusters on chromosomes 4 and 8 were excluded from subsequent analyses.

Fourteen QTL clusters had overlapping CIs and were thus confirmed as lodging QTL. Two of 14 clusters on chromosomes 9 and 10 were confirmed in different locations or water treatments. The rest of the clusters were confirmed in two or more mapping populations. Based on 95% CIs, six QTL of the H x C population were shared by one or more previous studies^18,19,22,34^. Four new QTL were identified on chromosomes 7 (one QTL) and 10 (three) under irrigated and rainfed field trials in both years.

We investigated whether QTL for stem-related traits, root-related traits, and node number were positioned in the CIs of lodging resistance QTL. A considerable number of QTL were found: QTL for stem strength^8^ (40.8, 106.8, 77.3, and 62.9 cM on chromosomes 4, 6, 13, and 14, respectively), stem diameter^40^ (76.5 cM on 19), root morphology^9^ (64.9 cM on 3), root dry weight^41,42^ (17.35 and 28.38 cM on 3), root lateral number^43^ (12.3 cM on 19), and node number^10,44,45^ (105.8, 31.4, and 61.0 cM on 6, 11, and 13, respectively). Additionally, a locus for major growth habit, *Dt1*^46^, was positioned at 78.6 cM on chromosome 19 regardless of whether the parental cross combination was *dt1*/*dt1*^33^ or *dt1*/*Dt1*^35^.

### Meta-QTL analysis

**Figure 2:**
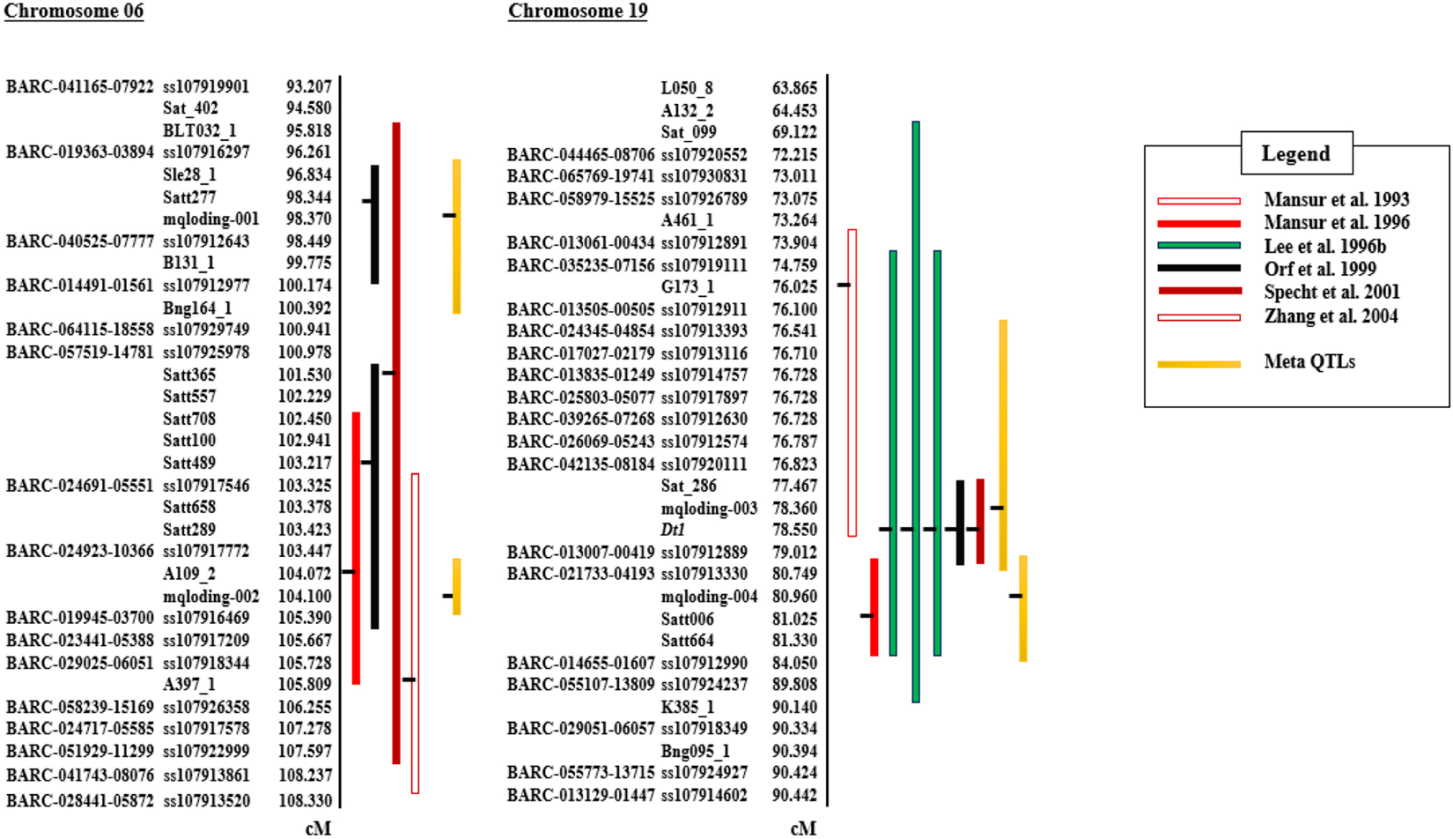
Meta-QTL for soybean lodging resistance. Four meta-QTL (yellow bars) were identified on two different chromosomes, 6 and 19. Positions of maximum likelihood-estimated QTL were determined (black horizontal bars). Colored vertical bars show the 95% confidence intervals. Major markers [either Beltsville Agricultural Research Center (BARC) local ID or SNP identifier in NCBI-dbSNP or both] that overlapped with or flanked QTLs were added to the left side of the chromosome. *cM* centimorgan.

Of the 14 QTL clusters, only two were used for meta-QTL analysis, as the others showed low likelihood of odd (LOD) values (or *P* values) in a single-marker analysis (SMA)^47^ due to insufficient design of the populations or QTL redundancy. A total of 12 QTL on chromosomes 6 (five QTL) and 19 (seven) were projected onto the consensus genetic map as a reference map for meta-analysis (Fig. 2). Across all QTL except for one on chromosome 6, the LOD values were greater than 2.5 (LOD 2.5 ≈ *P* < 0.001, assuming that the LOD or likelihood ratio (LR) asymptotically follows a χ^2^ distribution). The QTL on chromosome 6^48^ had a *P* value less than 0.01 (LOD = 1.75).

Five model selection criteria were used to determine how many meta-QTL could be chosen with the global likelihood value (Table 4). Four and five different mapping populations were used for meta-analysis of chromosomes 6 and 19, respectively. The 5 or 7 QTL were used to evaluate 1 to 5 or 1 to 7 meta-QTL in each QTL cluster. For a given number of meta-QTL, there were differences among the values of each model selection criterion. Except for the corrected Akaike information criterion (AICc), all model selection criteria for both QTL clusters showed the lowest value when two meta-QTL were used to estimate the maximum global likelihood value. When the AICc was used, this model selection criterion showed the lowest values for 3 meta-QTL and 1 meta-QTL on chromosomes 6 and 19, respectively. Considering all five model selection criteria values, the likelihood of two meta-QTL appeared to be the highest. Two QTL identified in the Minsoy x Noir 1 population (98.3 cM on chromosome 6^33^; 81.0 cM on 19^23^) were found to be significant clusters that contributed to the expected meta-QTL in a subsequent meta-analysis.

**Table 4:** Model selection for meta-QTL analysis of lodging on chromosomes 6 and 19.

Chromosome	Number of	Number of	Number of	Values of model selection criteria^d^ and delta (Δ)^e^									
	mapping populations^a^	QTL^b^	Meta-QTL^c^	AIC	AIC Δ^e^	AICc	AICc Δ	AIC3	AIC3 Δ	BIC	BIC Δ	AWE	AWE Δ
6	5	5	1	53.8	24.2	54.8	21.2	54.8	22.2	53.6	24.6	58.4	19.7
			2	29.6	0.0	41.6	8.0	32.6	0.0	30.0	0.0	38.7	0.0
			3	33.6	4.0	33.6	0.0	38.4	6.0	32.6	3.4	51.8	13.2
			4	.	.	.	.	.	.	.	.	.	.
			5	38.1	8.5	38.1	4.5	44.1	11.5	36.9	7.9	54.9	16.2
19	5	7	1	37.7	6.3	38.5	0.0	38.7	4.3	37.6	6.3	42.6	1.2
			2	31.2	0.0	39.3	0.9	34.4	0.0	31.2	0.0	41.4	0.0
			3	35.3	4.0	95.3	56.7	40.2	6.0	35.0	3.8	54.5	13.0
			4	39.3	8.0	39.3	0.8	46.3	12.0	38.9	7.7	68.2	26.8
			5	.	.	.	.	.	.	.	.	.	.
			6	.	.	.	.	.	.	.	.	.	.
			7	39.6	8.3	39.6	1.2	46.6	12.3	39.3	8.1	65.3	23.9

^a^This number indicates how many populations were used as independent populations for meta-analysis in each QTL cluster.

^b^The number of QTL indicates how many QTL in each QTL cluster.

^c^The optimal positions and number of meta-QTL were considered based on the number of QTL in each QTL cluster to test the best meta-QTL models.

^d^AIC, AICc (or AIC3), BIC, and AWE indicates Akaike information criterion, corrected Akaike information criterion, Bayesian information criterion, and approximate weight of evidence.

^e^This could be defined as the difference between model selection criteria values between two meta-QTL models such as the best meta-QTL model and other meta-QTL models.

Consequently, four meta-QTL were identified on chromosomes 6 and 19 (Fig. 2; Table 5). Two of them were located 5.73 cM apart on chromosome 6. There were no overlapping CIs between those with R^2^ values ranging from 0.15 to 0.21. Two meta-QTL on chromosome 19 showed slightly overlapping CIs with average R^2^ values from 0.27 to 0.39. Notably, all meta-QTL on chromosomes 6 and 19 were predicted major QTL, although meta-analysis generally decreased the CIs of meta-QTL.

**Table 5:** Meta-QTL for lodging resistance on chromosomes 6 and 10.

Chromosome	Designation of	Number of	Position of	Mean	meta-QTL^e^			
	meta-QTL^a^	meta-QTL^b^	meta-QTL^c^	R^2d^	Flaking markers			
					Left	Position	Right	Position
6	mqloding-001	2	98.370	0.21	Satt277	98.344	BARC-040525-07777	98.449
	mqloding-002		104.10	0.15	A109_2	104.072	BARC-019945-03700	105.390
19	mqloding-003	2	78.360	0.27	Sat_286	77.467	*Dt1*	78.550
	mqloding-004		80.960	0.39	BARC-021733-04193	80.749	Satt006	81.025
					**95% CI of meta-QTL** ^**e**^			
					**Flaking markers**			
					**Left**	**Position**	**Right**	**Position**
6	mqloding-001	2	98.370	0.21	BARC-019363-03894	96.261	BARC-064115-18558	100.941
	mqloding-002		104.100	0.15	BARC-024923-10366	103.447	BARC-019945-03700	105.390
19	mqloding-003	2	78.360	0.27	BARC-013505-00505	76.100	BARC-021733-04193	80.749
	mqloding-004		80.960	0.39	BARC-013007-00419	79.012	BARC-014655-01607	84.050

^a^The names of meta-QTL were designated for the purpose of using Soybase (https://soybase.org/).

^b^The number of meta-QTL was based on the values of five model selection criteria.

^c^The positions of meta-QTL were determined by the maximum joint likelihood values in the search for the best meta-QTL models. All positions were based on the Consensus 4.0 genetic map of soybean.

^d^The mean R^2^ values were averaged by the R^2^ values of QTL in a QTL cluster. However, in two meta-QTL, mqloding-001 and mqloding-004, because there was no QTL cluster, the R^2^ values were simply based on previous mapping results^23,33^.

^e^The positions and CIs of meta-QTL were based on the Consensus 4.0 genetic map of soybean.

## Discussion

We performed an integrative QTL study of the H x C population and previous mapping populations for lodging resistance, resulting in 14 significant QTL clusters. Meta-analysis of QTL identified in different studies located clusters of QTL on chromosomes 6 and 9.

There are apparent discrepancies in the QTL detected in different mapping studies, likely because of the type and number of markers incorporated, the population size, heritabilities of target QTL, QTL models, and linkage disequilibrium (LD)^49–51^. It has been suggested that meta-analysis could address such issues to achieve more efficient MAS^39^. Several QTL in previous populations were located in mapping intervals that span a wide range of CIs. Presumably, the main causes are population size, population type, and the false-positive level used for QTL confirmation. Considering the polymorphism information content (PIC)^52^, a large portion of genome segments in a backcross population may inherit monoallelic information. A small backcross population size could increase map distances between adjacent markers (typically over 20 cM), and the resulting larger map distances could overestimate R^2^ values and affect the estimation of CIs for QTL and meta-QTL. In addition, a low false-positive threshold could overestimate the additive effects of QTL and underestimate the CIs of QTL if those QTL originally had small gene effects^49^. Lander and Kruglyak^39^ demonstrated that false-positive QTL likely occur under stringent threshold values, such as the family-wise error rate (FWER). In our study, we identified meta-QTL in two QTL clusters in which the QTL mostly had stringent thresholds (LOD > 3.0) and overlapping CIs in multiple mapping populations. Thus, these meta-QTL can be appropriate consensus markers for MAS to improve lodging resistance.

A positive correlation between lodging and soybean maturity at the R8 growth stage was reported^22,23,25,26,53^. The correlation coefficient (γ) mostly ranged from 0.20 to 0.51. Our data from the H x C population showed a γ of 0.25 (*P* < 0.01). Our data and the independent studies were consistent with results deposited in Germplasm Resources Information Network (GRIN; http://www.ars-grin.gov/cgi-bin/npgs/html/site_holding.pl?SOY). In fact, the *E* series of loci, which are responsible for maturity or photoperiodism, were located in the CIs of QTL and QTL clusters for lodging. Historically, the maturity effect of the *E* series of loci was evaluated by genetic studies with near isogenic lines (NILs) of Harosoy or Clark^54^. *E1*^55,56^ and *E2*^55^ were positioned at 103.33 and 121.41 cM on chromosomes 6 and 10, respectively. Relative to *E2*, *E1* is particularly well known to have a major effect on flowering time, maturity, and branching^57,58^. *E3*, a type of phytochrome (*GmPhyA3*), was positioned at an interval between 78.26 and 94.5 cM on chromosome 19^59^. This interval included the QTL cluster on chromosome 19 revealed by our meta-analysis. A recent study indicated that allelic variation in *E1*, *E2*, *E3*, and *E4* explained approximately 64% of the phenotypic variation in flowering time among 63 soybean accessions^60^. *E5*^61^ and *E7*^62^ were closely located to *E2* and *E1*. Although *E4*^63^ (31.51 cM on chromosome 20) was not positioned in our study, these results indicate that lodging and maturity are fairly associated with each other.

We found several QTL for root and shoot traits were positioned in the CIs of lodging resistance QTL. Considering the effect of specific gene(s) (for example, *Dt1* and height), the relationships between lodging and other traits suggested that classical markers such as *E* and *Dt1* still had a strong and pleiotropic effect on lodging similar to that of a QTL cluster on chromosome 6.

Although recent QTL studies in plants are increasing our knowledge of the lodging resistance, the molecular mechanisms of lodging resistance remain unknown in most cases. Our study will further narrow down QTL intervals to provide resources for identification of candidate genes: for example, according to the current genome annotation Wm82.a2 (https://phytozome.jgi.doe.gov), there are 163 predicted genes (Glyma.02G293200 to Glyma.02G309400) within the interval to which mqloding-002 is mapped. To identify the sequence of candidate gene(s) from QTL intervals, the map-based cloning or sequence assembly within the intervals from the source of the lodging resistant soybean is needed. Further fine mapping to narrow down such intervals will also greatly facilitate the cloning of candidates.

Putative new lodging resistance QTL on chromosomes 7 and 10 were mapped in the H x C population. The growing number of QTL identified since the early 1990s, and the identification of new QTL demonstrates that there is still potential for discovering new lodging resistance QTL in soybean. Our effort will facilitate the identification of new resistance genes and QTL and will increase the pool of alleles that are important for the control of lodging. Since meta-analysis of QTL identified in different studies (experimental conditions/plant materials) can locate QTL more precisely, the four meta-QTL identified in our analysis will also aid in the development of improved markers to increase soybean breeding efficiency.

## Methods

### Population development

Two U.S. soybean (*Glycine max*) lines, Harosoy^64^ and Clark^65^, were used to develop populations. Harosoy and Clark were crossed as females and males, respectively. Each of eight F_1_ plants produced approximately 45 F_2_ seeds. The plant-to-row method was applied to advance the generation from F_2_ to F_6_. A total of 25 F_6_ plants in each of the 300 F_6_-derived rows were bulk threshed by row to generate 300 RILs. The number of RILs derived from each F_1_ ranged from 34 to 40. Parental lines of Harosoy [Mandarin (Ottawa) (2) x A.K. (Harrow)] or Clark [Lincoln (2) x Richland] were also propagated and harvested each year. These four parental lines are considered soybean ancestors that made significant contributions to the genetic composition of U.S. soybean cultivars.

### Genotyping of the population

Leaf tissue was collected in bulk from each of 300 rows of F_6_ individuals, eight rows of Harosoy or Clark individuals, and four rows of individuals of each parental line of Harosoy or Clark. DNA was diluted to 20 ng/μl. A modified protocol by Akkaya *et al*.^66^ was used for PCR. Most of the simple sequence repeat (SSR) amplicons were loaded onto a 2.5% agarose gel, and the differences among these were observed after electrophoretic separation at 70 V. Ambiguous SSR amplicons were loaded onto a polyacrylamide gel and separated in a 4300 DNA Analyzer with the LI-COR Saga V. 2 program (LI-COR Bioscience, Lincoln, NE). For single nucleotide polymorphism (SNP) genotyping, the Illumina GoldenGate Assay was performed with the 1536-SNP USLP 1.0 array^38,67^. GenCall software (Illumina, Inc., San Diego, CA) was used to identify allelic variation. Four classical markers including the pubescence color locus (*TT*/*tt*), hilum color locus (*RR*/*rr*), hilum color intensity locus (*II*/*i*^*i*^*i*^*i*^), and maturity date locus (*E2E2*/*e2e2*)^46^ were scored in 300 F_6_-derived RILs. Both Harosoy and Clark are homozygous (*W1W1*) for purple flower color. Clark (*TTRR*) is dominant over Harosoy (*ttrr*) in pubescence and hilum color. Harosoy (*II*) is dominant over Clark (*i*^*i*^*i*^*i*^) in hilum color intensity. Clark (*E2E2*) exhibited late maturity, whereas Harosoy (*e2e2*) displayed early maturity, indicating that late maturity is incompletely dominant over early maturity^46,55^. The genotypic values of only the RILs with definitively extreme maturity dates were scored in the H x C population.

### Genetic map construction

A total of 751 markers (4 classical markers, 266 SSRs, and 481 SNPs) were used for linkage analysis in the R/qtl software^68^. We examined linkage groups using the R/qtl function formLinkageGroups() [an initial value of a LOD was 15]. For linkage grouping, LOD value of 3.0 that established known linkage groups was chosen as the significance criterion for multipoint linkage testing. The maximum distance between two flanking markers was 0.372 rf (recombination fraction obtained with the Morgan function). The Kosambi mapping function^69^ was used for linkage analysis. To fix gaps in the genetic map, we set the maximum rf value to 0.450, which equaled the Kosambi map distance of 73.61 cM. To estimate linkage distances, a genotyping error of 0.01% was assumed^70^. Linkage distance was estimated by maximum likelihood (ML) with the expectation-maximization (EM) algorithm^71^. The default maximum iteration number was 10,000, and 0.000001 was used as the tolerance value. After checking the genotyping error^68^ (Supplemental Fig. 2), we finalized the H x C genetic map with 20 chromosomes and 730 markers (4 classical markers, 260 SSRs, and 466 SNPs) in 285 RILs. Given that our marker data type fits the framework of the Consensus 4.0 genetic map of soybean^38^ (all markers used in our study are present in the dataset used for the consensus map), we projected markers of our H x C population onto the consensus map to examine the relative marker positions.

### Field experiment

Lodging was visually rated on a scale of 1 (erect) to 5 (prostrate) when plants reached the R8 stage^72^. Field trials were carried out at the west field in East Campus, University of Nebraska-Lincoln Agronomy Research Farm, in 1998 (F_6:8_ plants) and 1999 (F_6:9_). The soil at the test site is a Sharpsburg silty clay loam. A two-replicate balanced incomplete block design (BIBD) was used to control for the effect of maturity. Sixteen blocks, each containing 20 RILs, were designed based on maturity date. Additionally, each block had one of the parental lines. All entries were planted into 2-row plots with 0.762 m row spacing and a length of 3.05 m, which resulted in a seeding rate of approximately 106,473 seeds/acre. Each plot had two replications under rainfed and irrigated conditions.

SAS (V. 9.0) was used for randomization, ANOVA, least square mean estimation, independent *t*-tests, normality tests, and heritability estimation. Two commands, ‘PROC UNIVARIATE’ and ‘PROC GPLOT’, were used for the normality test of lodging. Randomization was conducted by the ‘PROC PLAN’ command. ANOVAs were performed on the phenotypic data collected in each year and on the data across the years of 1998 and 1999. The ‘PROC MIXED’ model is as follow:

Y_*ijklm*_ = a_0_ + a_1_ × 1_*i*_ + a_2_ × 2_*j*_ + a_3_X3_*k*_ + a_4_X4_*l*_ + a_5_X5_*m*_ + a_6_X1_*i*_X2_*j*_ + a_7_X2_*j*_X5_*m*_ + a_8_X1_*i*_X5_*m*_ + a_9_X1_*i*_X2_*j*_X5_*m* + _ɛ_*ijklm*_ [Y: Lodging resistance, X1: Water treatment, X2: Year, X3: Replication, X4: Block (Replication), X5: RIL, X1 × 2: Water x Year interaction, X2X5: Year x RIL interaction, X1 × 5: Water x RIL interaction, X1X2 × 5: Water x Year x RIL interactions, a_0_: Overall mean, a_1_ ~ a_9_: Coefficient for each factor, ɛ: Error, which follows the normal distribution (mean: 0, variance: σ^2^_ε_)].

All classification variables except for the water treatment term were treated as random variables. The command ‘PROC MIXED (method = type3)’ was used for ANOVA and heritability estimation. Based on the mixed model ANOVA results, least square (LS) means were estimated with the command ‘PROC LSMEANS’. The LS means for each water-year data set were used for QTL analysis. The CIs of heritability were estimated by progeny means basis^73^. The correlation coefficients between lodging and other traits were estimated with the command ‘PROC CORR’. A 5% false-positive value was used for all statistical tests as the α level criterion.

### QTL analysis

An ML approach^74^ with the EM algorithm^75^ was used to estimate parameters for composite interval mapping (CIM)^76^ with a single-QTL model. The null hypothesis (H_o_) for CIM was that there was no QTL anywhere in the genome. The alternative hypothesis (H_a_) was that there was a QTL in an interval between flanking markers. WinQTL Cartographer V. 2.5 software^77^ was used for the QTL analysis, which was applied to the data set of 285 RILs with the 751 combined classical, SSR, and SNP markers. A permutation test (10,000 repeats)^78^ was applied to empirically determine the critical LR value for declaring the existence of a QTL. The LR statistic approximately followed a χ^2^ distribution. Based on the ANOVA result, LR threshold values were estimated based on each water-year data set. In WinQTL Cartographer V. 2.5, model 6 was chosen to conduct a CIM analysis on the H x C data set. Up to five markers were used as control background markers. For background marker selection, the stepwise selection method was used to mitigate errors arising from using only a forward or only a backward selection approach. To avoid having too many background markers (model overfitting), an alpha value of 0.05 was used for both the forward and backward regression. A window size of 1 cM on either side of the interval flanked by a marker pair was chosen to control background marker effects and to generate an LOD profile across the whole genome with a default window size of 10. To separate multiple close peaks above the LR threshold in WinQTL Cartographer, the parameters for the single-QTL model included a minimum distance of 5 cM between the putative map positions of adjacent putative QTL. The LOD peak values, additive effects, and 95% CIs were estimated based on the ML-estimated QTL positions. An LOD score profile was generated using a 1 cM walking speed.

### Data mining and QTL projection

All QTL mapping information was collected from the public database Soybase (http://soybase.org) (Table 3 and Supplemental Table 2). For QTL confirmation and meta-analysis, we collected QTL marker positions, R^2^ values, the size of the population, and CIs from previous studies. CIs of previous QTL in independent studies were calculated as follows:

$$95 \% \,{\rm{CI}}=530/({\rm{N}}\times {{\rm{R}}}^{2})\,{\rm{for}}\,{\rm{a}}\,{\rm{backcross}}\,{\rm{or}}\,{\rm{an}}\,{{\rm{F}}}_{{\rm{2}}}\,{\rm{population}}$$

$$\mathrm{95} \% \,{\rm{CI}}=\mathrm{163}/(N\times {{\rm{R}}}^{{\rm{2}}})\,{\rm{for}}\,{\rm{an}}\,{\rm{RIL}}\,{\rm{population}}$$

where R^2^ was the phenotypic variance explained by a QTL and N was the size of the population. QTL with 95% CIs were projected onto the Consensus 4.0 genetic map of soybean for QTL confirmation^70^.

### Meta-analysis

For meta-analysis, QTL were projected onto the Consensus 4.0 genetic map of soybean with BioMercator V. 3.0^79^ and an algorithm from MetaQTL^80^. QTL clusters, which included the overlapping 95% CIs of two or more QTL, were used to identify the CIs and positions of meta-QTL. The assumption of the meta-analysis was that the variance of the ML-estimated QTL positions followed a normal distribution. The variance of each projected QTL position in a QTL cluster was estimated from the 95% CI^51^ as follows:

$$\mathrm{95} \% \,{\rm{CI}}={\rm{3.92}}\times {\rm{Variance}}\,{({\rm{QTL}})}^{1/2}$$

The variance of a meta-QTL position was estimated by the formula:

$${\rm{Variance}}\,(\mathrm{QTL})=1/\sum _{i=1}^{n}\frac{1}{{\rm{Variance}}\,(\mathrm{QTL}i)}$$

where Variance (QTL_*i*_) is the variance of the _*i*_th QTL in a QTL cluster with *n* QTL. In a window size based on the CIs of QTL in a QTL cluster, ML values were obtained with the meta-analysis models. To determine whether there is more than one meta-QTL in each QTL cluster, we evaluated from 1 to *n* (where *n* is the number of meta-QTL in each QTL cluster) meta-QTL models using joint-mixture normal functions^81^. We evaluated the meta-QTL models using five criteria: Akaike information criterion (AIC), corrected Akaike information criterion (AICc and AIC3), Bayesian information criterion (BIC), and approximate weight of evidence (AWE). Using the most appropriate model from these selection criteria, we identified the consensus meta-QTL positions. QTL identified in at least two independent field experiments (either location or year) were used for meta-analysis. If there were multiple QTL in the same field experiment, we chose one with a high LOD or R^2^ value. If a QTL was identified by using means across locations and/or years, it was considered to be from an independent experiment.

## Supplementary information

Dataset 1
