# Supplementary material for: Integration of lodging resistance QTL in soybean
Source: Sci Rep. 2019 Apr 25;9:6540. doi: 10.1038/s41598-019-42965-6 (PMC6484036; doi:10.1038/s41598-019-42965-6)
Supplement: Supplementary file 1 — Dataset 1 [file 41598_2019_42965_MOESM1_ESM.pdf]

Integration of lodging resistance QTL in soybean

Sadal Hwang[1] and Tong Geon Lee

Supplemental Table 1**.** Analysis of variance for lodging in the Harosoy x Clark population.

Source	*d.f.*[a]	MS[b]	F-value	*Pr* > F
Water	1	21.5651	0.36	0.6577
Year	1	40.9509	0.67	0.5618
Year x Water	1	60.6426	167.29	< 0.0001
Replication	1	0.0037	0.01	0.9365
Block(Replication)	15	0.5678	2.24	0.0043
RIL	284	2.2383	3.49	< 0.0001
Year x RIL	299	0.6240	1.72	< 0.0001
Water x RIL	299	0.3803	1.05	0.3393
Year x Water x RIL	299	0.3625	1.43	< 0.0001
Error	1184	0.2536		

[a] *d.f.*, degree of freedom.
[b] *MS*, mean square.

Supplemental Table 2. Previous QTL data for lodging.

Reference	Chr.	QTL marker[a]	Position	LOD or *P* value	R^2	Flanking markers[b]		Field location and/or year
Mansur et al. 1993	19	G173_1	76.025	LOD = 8.9	0.45	A461_1	BARC-013007-00419	Ames, IW, 1991-1992
Mansur et al. 1996	06	A109_2	104.072	*P* ≤ 0.0010	0.17	Satt557	Satt557	-
Mansur et al. 1996	16	A060_1	26.359	*P* ≤ 0.0010	0.06	A204_1	BARC-029477-06200	-
Mansur et al. 1996	19	BLT007_1	8.653	*P* ≤ 0.0010	0.07	Sle3_4s	Satt182	-
Mansur et al. 1996	19	Satt006	81.025	*P* ≤ 0.0010	0.27	BARC-013007-00419	BARC-014655-01607	-
Lee et al. 1996a	04	BARC-015121-02570	100.565	*P* = 0.0080	0.07	Bng012_1	A074_1	Combined[c]
Lee et al. 1996a	04	BARC-013699-01240	97.433	*P* = 0.0240	0.05	Bng012_1	BARC-032045-07244	Combined
Lee et al. 1996a	08	A117_1	91.877	*P* = 0.0020	0.09	BARC-031601-07118	BARC-057653-14889	Combined
Lee et al. 1996a	08	BARC-055945-13878	102.181	*P* = 0.0310	0.05	BARC-014453-01374	BARC-028975-06047	Combined
Lee et al. 1996a	09	BARC-039101-07449	69.997	*P* = 0.0240	0.05	Sct_190	BARC-030025-06777	Combined
Lee et al. 1996a	13	Sat_197	77.324	*P* = 0.0230	0.06	Satt362	A566_1	Combined
Lee et al. 1996a	13	K014_2	82.094	*P* = 0.0440	0.04	BARC-013257-00462	BARC-025915-05157	Combined
Lee et al. 1996a	15	BARC-014819-01679	35.006	*P* = 0.0480	0.09	A636_1	*B2*	Combined
Lee et al. 1996a	15	A597_1	71.128	*P* = 0.0190	0.05	A646_1	A226H_2	Combined
Lee et al. 1996a	15	BLT049_5	77.746	*P* = 0.0410	0.04	A646_1	Satt231	Combined
Lee et al. 1996a	18	L183_1	104.419	*P* = 0.0090	0.07	BARC-010255-00571	A681_1	Combined
Lee et al. 1996a	18	A378_1	101.823	*P* = 0.0160	0.06	Satt191	A681_1	Combined
Lee et al. 1996a	19	A169_1	0.902	*P* = 0.0010	0.13	BARC-060295-16596	BLT010_2	Combined
Lee et al. 1996a	19	BARC-039375-07304	3.362	*P* = 0.0010	0.12	BARC-060295-16596	Sat_301	Combined
Lee et al. 1996b	19	*Dt1*	78.550	*P* ≤ 0.0100	0.53	BARC-013061-00434	BARC-014655-01607	Combined
Lee et al. 1996b	19	*Dt1*	78.550	LOD = 18.3	0.56	BARC-013061-00434	BARC-014655-01607	Combined
Lee et al. 1996b	19	*Dt1*	78.550	LOD = 4.5	0.21	A132_2	K385_1	Athens, GA
Lee et al. 1996b	19	*Dt1*	78.550	LOD = 18.3	0.56	BARC-013061-00434	BARC-014655-01607	Blackville, SC
Orf et al. 1999	01	Sat_036	57.517	LOD = 3.2	0.06	Mng474_2	K019_1	Rosemount, MN
Orf et al. 1999	02	Sat_096	10.832	LOD = 5.0	0.10	BARC-025791-05070	BARC-022263-04301	Los Andes, Chile

Orf et al. 1999	06	Satt277	98.344	LOD = 12	0.21	BARC-019363-03894	BARC-014491-01561	Rosemount, MN
Orf et al. 1999	06	Satt489	103.217	LOD = 8.1	0.15	BARC-064115-18558	BARC-023441-05388	Rosemount, MN
Orf et al. 1999	13	Satt335	61.053	LOD = 9.2	0.17	BARC-900492-00930	BARC-061189-17109	Waseca, MN
Orf et al. 1999	19	Satt182	13.074	LOD = 4	0.08	BLT007_1	BARC-065445-19463	Rosemount, MN
Orf et al. 1999	19	*Dt1*	78.550	LOD = 16	0.27	BARC-042135-08184	BARC-021733-04193	Rosemount, MN
Specht et al. 2001	06	Satt365	101.530	LOD = 6.5	0.06	Sat_402	BARC-051929-11299	-
Specht et al. 2001	12	*Ps*	76.766	LOD = 6.2	0.05	Sat_158	BARC-026145-05280	-
Specht et al. 2001	19	Satt232	12.346	LOD = 13.3	0.13	BLT007_1	BARC-065445-19463	-
Specht et al. 2001	19	L050_7	38.811	LOD = 4.5	0.04	BARC-059443-15806	peG488_1	-
Specht et al. 2001	19	*Dt1*	78.550	LOD = 25.8	0.27	BARC-042135-08184	BARC-021733-04193	-
Chapman et al. 2003	11	Satt251	38.802	$P < 0.050$	0.04	BARC-014611-01591	Sat_247	Springfield, TN
Wang et al. 2004	09	Satt137	32.300	LOD = 13.1	0.29	BARC-028040-06720	Satt518	-
Zhang et al. 2004	06	A397_1	105.809	LOD = 8.3	0.18	BARC-024691-05551	BARC-028441-05872	Nanjing, China
Zhang et al. 2004	06	Satt319	103.199	LOD = 7.2	0.15	BARC-014491-01561	BARC-058239-15169	Nanjing, China
Zhang et al. 2004	13	Satt114	43.898	LOD = 3.2	0.05	BARC-023255-03843	B212_1	Nanjing, China
Kabelka et al. 2004	02	Satt542	61.840	LOD = 2.6	0.06	BARC-025183-06457	BARC-047945-10443	-
Reinprecht et al. 2006	17	Satt311	72.522	LOD = 2.0	0.05	BARC-013969-01290	K011_5	Ridgetown, Ontario, Canada, 2000
Reinprecht et al. 2006	18	Satt324	35.428	LOD = 3.5	0.10	BARC-018775-03015	BARC-063985-18522	Harrow, Ontario, Canada, 2000
Reinprecht et al. 2006	11	Sat_123	96.149	LOD = 2.5	0.07	Satt444	L050_1	Woodslee, Ontario, Canada, 2001
Reinprecht et al. 2006	01	Satt071	82.883	LOD = 2.6	0.06	BARC-008013-00215	BARC-018835-03260	Woodslee, Ontario, Canada, 2001
Reinprecht et al. 2006	19	Satt182	13.074	LOD = 3.9	0.12	BLT007_1	BARC-065445-19463	Ridgetown, Ontario, Canada, 2001
Guzman et al. 2007	18	Satt191	89.372	$P < 0.0012$	0.14	Sat_143	A681_1	Columbus, OH, 2003
Guzman et al. 2007	18	Satt191	89.372	$P < 0.0010$	0.27	BARC-059017-15576	BARC-062769-18043	West Lafayette, IN, 2004
Guzman et al. 2007	14	Satt534	75.727	LOD = 2.0	0.14	Satt168	Tel450	Columbus, OH, 2003
Guzman et al. 2007	03	Satt485	28.389	LOD = 2.1	0.13	*L2*	*S11*	West Lafayette, IN, 2004
Guzman et al. 2007	14	Satt126	23.286	LOD = 2.1	0.12	BLT013_2	A329_1	Columbus, OH, 2003
Guzman et al. 2007	03	Satt257	74.707	$P = 0.0094$	0.09	Satt125	A363_3	Combined
Guzman et al. 2007	19	Satt232	12.346	LOD = 6.7	0.34	Satt723	B164_1	West Lafayette, IN, 2004
Panthee et al. 2007	05	Satt225	79.303	LOD = 3.9	0.21	Bng017_1	BARC-020301-04546	Combined
Panthee et al. 2007	05	Satt593	24.713	LOD = 5.2	0.33	BARC-052043-11321	Satt526	Combined
Li et al. 2008	20	Satt330	70.117	LOD = 2.2	0.07	BARC-017311-02261	BARC-029301-06148	Lexington, KY, 2004

Li et al. 2008	20	Satt330	70.117	LOD = 3.4	0.10	Satt049	BARC-041155-07919	Princeton, KY, 2003
Li et al. 2008	20	Satt330	70.117	LOD = 3.0	0.09	BARC-053115-11717	BARC-042685-08348	Princeton, KY, 2004
Li et al. 2008	19	Satt284	33.938	LOD = 3.4	0.10	Satt182	Sat_340	Lexington, KY, 2004
Li et al. 2008	19	Satt284	33.938	LOD = 3.4	0.10	Satt182	Sat_340	Princeton, KY, 2003
Li et al. 2008	19	Satt284	33.938	LOD = 6.7	0.19	B164_1	B162_2	Princeton, KY, 2003
Li et al. 2008	03	Satt549	57.265	LOD = 2.9	0.09	Sat_208	BARC-048557-10665	Lexington, KY, 2004
Li et al. 2008	03	Satt549	57.265	LOD = 2.8	0.08	Satt125	BARC-061771-17371	Princeton, KY, 2003
Palomeque et al. 2009	09	Sat_126	91.198	$P = 0.0041$	0.10	Sat_020	Bng007_1	Woodstock, 2005
Palomeque et al. 2009	09	Sat_126	91.198	$P < 0.0001$	0.19	BARC-042081-08175	E014_2	Ontario, 2005
Palomeque et al. 2010	20	Satt162	78.817	$P = 0.0028$	0.09	BARC-022381-04318	BARC-042897-08454	Ottawa, 2006
Kim et al. 2012	04	BARC-039915-07604	6.745	LOD = 1.76	0.07	Sct_186	Satt578	-
Kim et al. 2012	18	BARC-051587-11167	44.053	LOD = 2.7	0.13	BARC-003432-00279	BARC-027694-06635	-
Rossi et al. 2013	04	Satt139	49.785	$P < 0.0100$	0.13	BARC-031795-07219	BARC-052885-11664	Ontario, Canada
Rossi et al. 2013	06	Satt277	98.344	$P < 0.0100$	0.17	BARC-041165-07922	BARC-024923-10366	Ontario, Canada
Rossi et al. 2013	06	Satt100	102.94 1	$P < 0.0100$	0.19	Satt277	BARC-051929-11299	Ontario, Canada
Rossi et al. 2013	14	Satt126	23.286	$P < 0.0100$	0.13	BARC-049217-10824	BARC-007338-00165	MN, USA
Rossi et al. 2013	02	Satt005	83.414	$P < 0.0100$	0.09	Sat_423	BARC-055839-13759	Ontario, Canada
Rossi et al. 2013	13	Sat_120	58.425	$P < 0.0100$	0.11	R045_1	BARC-061189-17109	MN, USA
Lee et al. 2015	04	BARC-038359-10052	5.442	LOD = 3.8	0.13	Sct_186	BARC-024117-04765	Combined
Lee et al. 2015	19	BARC-044415-08701	55.881	LOD = 3.3	0.11	p28_10_2	A132_2	Combined
Lee et al. 2015	04	BARC-044691-08761	32.272	$P < 0.0010$	0.14	BARC-015463-01971	BARC-031123-07000	-
Lee et al. 2015	19	BARC-041643-08051	25.845	$P < 0.0030$	0.09	Satt182	BARC-031583-07115	-

[a] Proximate markers were indicated when marker information were not available (i.e., Lee et al. 1996a).
[b] The flanking markers were based on the 95% CIs calculated in the current study.
[c] QTL information were identified across locations and/or years.

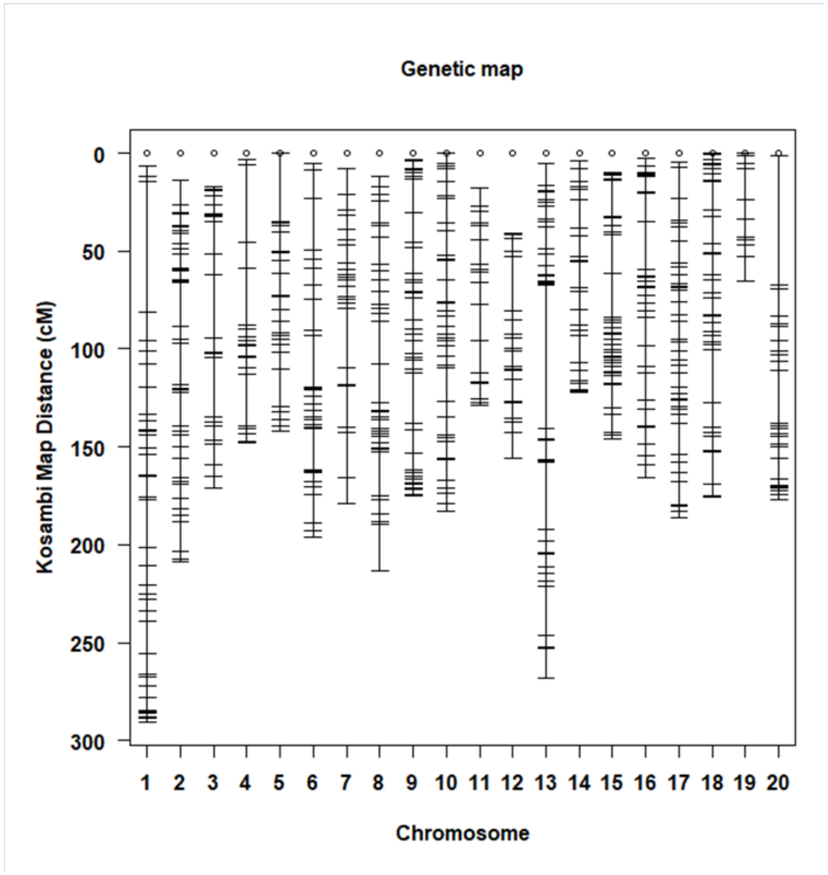

Supplemental Fig. 1 Genetic map of the Harosoy x Clark (H x C) population.

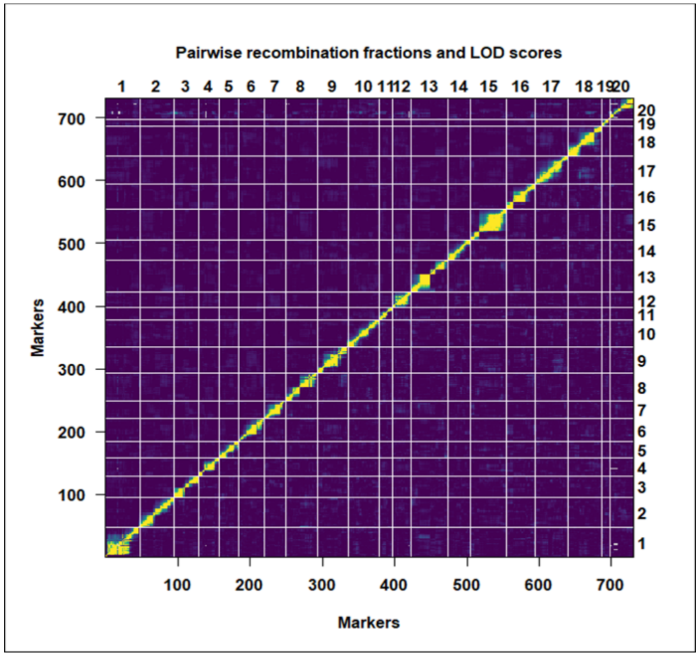

Supplemental Fig. 2 Plot of estimated pairwise recombination fraction and likelihood of odd values for linkage among markers.
